# Systematic Techniques to Enhance rEtention in Randomised controlled trials: the STEER study protocol

**DOI:** 10.1186/s13063-018-2572-0

**Published:** 2018-03-27

**Authors:** Katie Gillies, Peter Bower, Jim Elliott, Graeme MacLennan, Rumana S. N. Newlands, Margaret Ogden, Shaun P. Treweek, Mary Wells, Miles D. Witham, Bridget Young, Jill J. Francis

**Affiliations:** 1 Health Services Research Unit, 3rd Floor Health Sciences Building, Institute of Applied Health Sciences, School of Medicine, Medical Sciences and Nutrition, University of Aberdeen, Foresterhill, Aberdeen, AB25 2ZD UK; 2 MRC North West Hub for Trials Methodology Research, Manchester Academic Health Science Centre, University of Manchester, Oxford Road, Manchester, M13 9PL UK; 3 The Centre for Healthcare Randomised Trials (CHaRT), 3rd Floor Health Sciences Building, Institute of Applied Health Sciences, School of Medicine, Medical Sciences and Nutrition, University of Aberdeen, Foresterhill, Aberdeen, AB25 2ZD UK; 4 Health Services Research Unit, 2nd Floor Health Sciences Building, Institute of Applied Health Sciences, School of Medicine, Medical Sciences and Nutrition, University of Aberdeen, Foresterhill, Aberdeen, AB25 2ZD UK; 5 NMAHP Research Unit, University of Stirling, Stirling, FK9 4LA UK; 6 Ageing and Health, University of Dundee, Ninewells Hospital, Dundee, DD1 9SY UK; 7 Department of Psychological Sciences, Institute of Psychology, Health and Society, Whelan Building, University of Liverpool, Brownlow Hill, Liverpool, L69 3GB UK; 8 School of Health Sciences, City, University of London, Northampton Square, London, EC1V 0HB UK

**Keywords:** Trials, Retention, Non-retention, Dropout, Theory, Intervention, Interviews

## Abstract

**Background:**

Non-retention of participants seriously affects the credibility of clinical trial results and significantly reduces the potential of a trial to influence clinical practice. Non-retention can be defined as instances where participants leave the study prematurely. Examples include withdrawal of consent and loss to follow-up and thus outcome data cannot be obtained. The majority of existing interventions targeting retention fail to describe any theoretical basis for the observed improvement, or lack of improvement. Moreover, most of these interventions lack involvement of participants in their conception and/or design, raising questions about their relevance and acceptability. Many of the causes of non-retention involve people performing a behaviour (e.g. not returning a questionnaire). Behaviour change is difficult, and the importance of a strong theoretical basis for interventions that aim to change behaviour is increasingly recognised. This research aims to develop and pilot theoretically informed, participant-centred, evidence-based behaviour change interventions to improve retention in trials.

**Methods:**

This research will generate data through semi-structured interviews on stakeholders’ perspectives of the reasons for trial non-retention. It will identify perceived barriers and enablers to trial retention using the Theoretical Domains Framework. The intervention development work will involve identification of behaviour change techniques, using recognised methodology, and co-production of retention interventions through discussion groups with end-users. An evaluation of intervention acceptability and feasibility will be conducted in focus groups. Finally, a ready-to-use evaluation framework to deploy in Studies Within A Trial as well as an explanatory retention framework will be developed for identifying and tackling modifiable issues to improve trial retention.

**Discussion:**

We believe this to be one of the first studies to apply a theoretical lens to the development of interventions to improve trial retention that have been informed by, and are embedded within, participants’ experiential accounts. By developing and identifying priority interventions this study will support efforts to reduce research waste.

**Electronic supplementary material:**

The online version of this article (10.1186/s13063-018-2572-0) contains supplementary material, which is available to authorized users.

## Background

Randomised controlled trials are the cornerstone of evidence-based healthcare as they provide unbiased estimates of the benefits and harms of treatment if conducted rigorously [1]. It is common for many trial participants (sometimes more than 20%) to drop out before the trial finishes [2]. Moreover, 50% of trials have loss to follow-up of over 11% [2]. Dropout is defined here as non-retention as per the ‘Standard Protocol Items: Recommendations for Interventional Trials’ (SPIRIT) guidelines. SPIRIT defines non-retention as ‘instances where participants are prematurely “off-study” (i.e., consent withdrawn or lost to follow-up) and thus outcome data cannot be obtained from them’. Non-retention, therefore, seriously affects the credibility of trial results and significantly affects the potential of a trial to influence clinical practice [3]. Recent estimates have shown that the results of around half of all clinical trials could have been overturned if the outcomes from the non-retainers were known [4]. In other words, healthcare systems may not be delivering the best possible care because trial dropout has undermined the evidence upon which that care choice is based. Our research aims to develop and test participant-centred, theoretically informed interventions to reduce non-retention in trials using insights from behaviour change theory. These interventions have the potential to improve the evidence base on which clinical care treatment choices are made.

Much current research on interventions to minimise non-retention is not based on theory or formal evidence [5]. Yet many aspects of non-retention involve performing a behaviour (e.g. returning a questionnaire) and may be influenced and modified by a range of factors (e.g. attending a follow-up clinic may be influenced by access to transport). The Cochrane review on trial retention strategies provides little guidance to improve retention beyond offering money to return questionnaires; there were no included interventions designed to target the reasons why people may drop out of trials [5]. Without effective ways of reducing non-retention, trials currently have to build in inflated sample sizes at baseline to allow for trial participants who will not be retained. The average cost of a clinical trial in the UK is about £8500 per participant [6]; so recruiting extra participants to account for non-retention, therefore, imposes significant costs on trial funders – costs which are not then available to support additional trials. Not surprisingly, finding evidence-based ways to reduce non-retention has been identified as one of the top three research priorities by the UK clinical trial community [7].

The majority of existing interventions targeting retention fail to describe any theoretical, and hence generalisable, basis for the observed improvement, or lack of improvement [5]. Moreover, most of these interventions lack involvement of participants in their conception and/or design, raising questions about their relevance and acceptability [5]. Many of the causes of non-retention involve people performing, or not performing, an action such as returning a questionnaire; in other words, performing a behaviour. Behaviour change is difficult, and the importance of a strong theoretical basis for interventions that aim to change behaviour is increasingly recognised [8]. Framing intervention design with reference to behavioural theory offers the possibility of a rationale for developing targeted interventions to support retention and, moreover, provides the basis for a widely applicable methodology for designing such interventions [8]. Evidence from one trial evaluating a theoretically informed letter to target return of trial questionnaires showed a 6% improvement in response rates in the intervention group [9]. However, the perspectives of people in the target group (i.e. potential non-retainers) were not incorporated into the design of the intervention suggesting that the potential benefit may be even larger were these perspectives taken into consideration during design. The research described in this protocol will combine the use of behavioural theory and participant perspectives to underpin retention intervention development and directly address this evidence gap.

Before developing interventions to change behaviour it is important to define the behaviour of interest. For the purposes of the research described in this protocol the behaviour of interest is non-retention relating to failure to collect trial primary outcome data. To define this further, ‘trial non-retention’ encompasses several specific aspects of behaviour, namely:

- Failure to return questionnaire data (where mode of delivery/response may vary from postal, email or others)
- Failure to attend follow-up clinics as part of trial follow-up procedures
- Participant request for no further follow-up

Applying the Target, Action, Context, Time, Actor (TACTA) framework to the behaviour, the behaviour would be specified as follows [10]:

- Target: all trial participants
- Action: e.g. return of questionnaire or attendance at clinic
- Context: e.g. at home or in clinic
- Time: e.g. dependent on trial follow-up time points
- Actor: e.g. trial study staff or trial site staff or clinical staff

This research will not be considering non-adherence to trial interventions or failure to correctly complete questionnaires (e.g. return of questionnaire but missing primary outcome data) as these are outside the scope of this work. The reason for not including these behaviours in this research is that the nature of the underlying behaviour is likely demonstrably different to that of non-retention and, therefore, would require a different focus for intervention development.

**Fig. 1:**
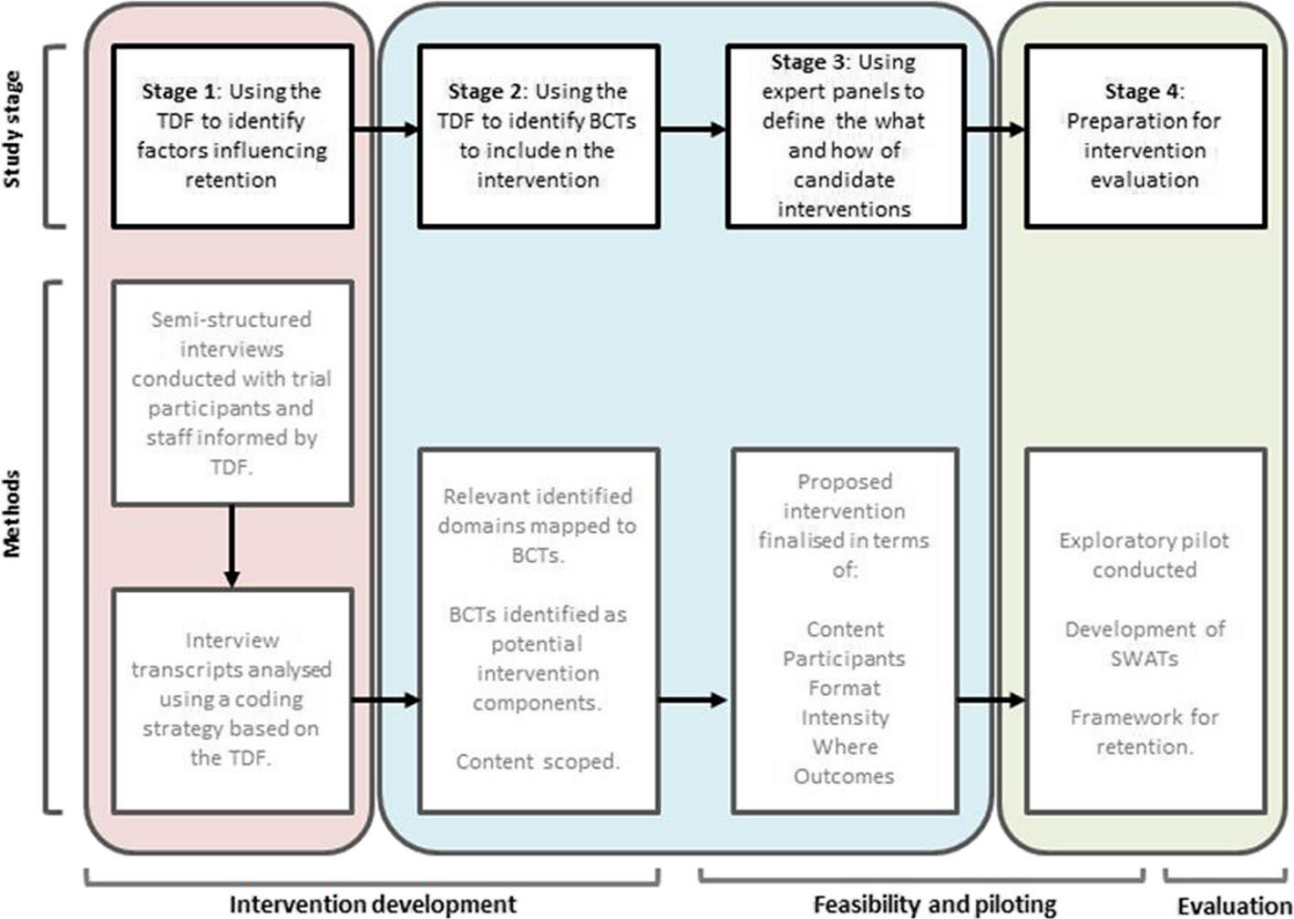
STEER protocol overview

This protocol details research that aims to develop theoretically informed, participant-centred, retention interventions (see Fig. 1) The structure of the work is based on the methodological approach recommended by the Medical Research Council (MRC) guidance for developing and evaluating complex interventions [11]. The MRC guidance provides a systematic approach to inform: intervention development; feasibility and piloting; evaluation (including process evaluation) and implementation. The research described in this protocol explicitly addresses intervention development, feasibility and piloting, and plans for the evaluation components of the guidance but will also explore initial perspectives on implementation. The project team will work with a range of stakeholders (defined as trial participants, and trial staff with direct insights about why participants fail to be retained, e.g. research nurses, trial managers, data coordinators) across various aspects of the project. Alongside the MRC framework for developing and evaluating complex interventions, the overarching methodology for the study will use an established theoretical framework (Theoretical Domains Framework, TDF) to inform the development of targeted retention interventions [12]. The TDF is a comprehensive framework that proposes 14 behaviour domains that may influence behaviour (e.g. knowledge, behavioural regulation, emotion). Priority domains are ascertained and context-specific barriers within each domain are identified, often by coding stakeholder interview data to identify barriers that are important to stakeholders, which are then targeted when developing behavioural interventions [13]. Key behaviour change techniques (BCTs) can be incorporated into the development of interventions to specifically target theoretical constructs. BCTs are defined as the smallest ‘active ingredient’ of an intervention. In other words, techniques that cannot be reduced further to smaller components, e.g. goal setting or self-monitoring of behaviour, and they can be used alone or in combination with other BCTs [14, 15]. The BCT taxonomy, generated through international expert consensus, contains 93 techniques that can be applied to behaviour change interventions to systematically describe, review and replicate core content and can also be used to develop new, complex, behaviour change interventions [14, 15].

Whilst some retention challenges will be common across trials, it is likely that others will be unique to particular trial problems and trial contexts. For the purposes of this project, we are defining context as ‘*the key features of the environment in which the work is immersed and which are interpreted as meaningful to the success, failure and unexpected consequences of the trial, as well as the relationship of these to the stakeholders*’ (adapted from Ogrinc et al.) [16]. To maximise transferability of findings from this research, trial context will be limited to Phase III pragmatic effectiveness randomised controlled trials (RCTs) that include adults who have capacity to consent for themselves in non-emergency settings. This research aims to deliver both (1) potential solutions for common retention challenges and (2) a generalisable method (i.e. development of behaviourally focussed retention interventions) for identifying and managing challenges unique to a given trial. The impact of this strategy will be to reduce the research waste associated with the common problem of substantial trial non-retention.

### Aim

The overall aim of the study is to develop and pilot theoretically informed, participant-centred, evidence-based behaviour change interventions to improve retention in trials.

### Research objectives (RO)

1. *Identify the problem* (identify stakeholders’ views and experiences of non-retention to determine ‘who needs to do what differently?’)
2. *Assess the problem* (use a theoretical framework to identify which barriers and enablers to retention in trials need to be targeted)
3. *Form possible solutions* (which behaviour change techniques could overcome the modifiable barriers and enhance the enablers of trial retention and how best could these techniques be presented); and
4. *Evaluate feasibility and acceptability of the selected interventions* (*e*xplore the feasibility and acceptability of potential interventions from the perspective of stakeholders and how retention interventions can be implemented in new and ongoing trials).

## Methods

### Identifying and assessing the problem (RO1 and RO2)

Research objectives 1 and 2 will generate data on stakeholders’ perspectives on the reasons for trial non-retention and identify perceived barriers and enablers to trial retention. Exploring these issues across a range of trials will allow us to draw conclusions about the barriers and enablers of retention likely to apply across trials as well as those that are more context specific.

#### Study design

We will conduct semi-structured interviews to explore participants’ experiences of the trial, non-retention, what could have been done to retain them or what helped them to continue and what behavioural barriers and enablers were experienced. To achieve this, adult participants who have dropped out or considered dropping out from on-going trials (identified from Aberdeen, Dundee and Manchester clinical trial unit portfolios) will be invited to participate. Previous published research (including work from our team) has successfully engaged people who drop out from trials in qualitative research [17–21].

In addition, trial staff will be interviewed to explore perceptions about why participants fail to be retained and what helped to keep others engaged in a specific trial, but also explore more generally what strategies or factors contribute to ‘good’ retention in trials. Interview guides will also investigate which behaviours (actions and non-actions) influenced trial non-retention.

#### Participant sampling and recruitment

##### Host trials

We will select trials with poor retention (e.g. those with more than 15% missing primary outcome data) from the portfolios of the trials units involved in this proposal. As much as possible, trials will be selected purposively to maximise variability in trial intervention and population (e.g. young vs old, multimorbidity vs little comorbidity, acute vs chronic conditions, etc.) such that influence of context is maximised in the sampling frame and data generated takes this into account. Specifically, we will sample trials for inclusion based on early vs late non-retention (which will be defined for each trial included in the sample but will likely be linked to data collection time points) and the potential influence of trial burden (i.e. how much ‘work’ is involved for the participant, e.g. through number of visits or questionnaires required to collect follow up data). Moreover, we will also consider ‘trial team expertise’. We will use the number of years of trial experience within the trial team (both for clinical and non-clinical team members) as factors to inform sampling. Wherever possible, included trials will be in the active follow-up phase such that the non-retention event is closely situated to the time of interview (i.e. no more than 3 months from attempt of last follow-up).

##### Sampling of participants

We will develop a purposive sampling strategy to identify trial participants who are not retained, or who deliberated about non-retention (identified by contacting retained participants or those participants who have missed one or more data collection time points but who subsequently re-engaged), from the trials identified above. We will maximise efforts to ensure that the participants invited to take part in this study are representative of those who are not retained in trials. Specifically, efforts will be made to maximise the diversity of the sample by identifying and inviting participants from all trial arms and across a range of socio-demographic backgrounds. In addition to trial participants, we will identify trial staff (e.g. research nurses, trial managers, data coordinators) associated with the poorly retaining trials and invite them for interview. Through purposive sampling a diverse range of participants (both trial participants and staff) will be included. Our aim is to recruit participants with a wide variety of trial experience and diverse perspectives on features that are important to people when deciding whether or not to be retained within an RCT.

##### Recruitment of participants

We will send an invitation letter and Participant Information Leaflet to prospective participants, both of which will be informed for content by our patient and public involvement (PPI) members, by post or by email, depending on the contact details held for participants. All trial staff will be contacted by email. Interested parties will be asked to contact the STEER research fellow (RN) directly to arrange a convenient time to conduct the interview. Two attempts will be made to engage with potential participants after which point no further contact will be made. Before the interview commences the researcher (RN) will obtain verbal informed consent from participants. The interviews will be conducted over the telephone so as to allow inclusion of participants across a wide geographic area but, where possible, face-to-face interviews will be offered.

We will conduct approximately 45 interviews (approximately six participants and three staff (e.g. research nurses, trial managers, data coordinators) from each of five on-going trials) as per sampling informed by similar studies [21]. Recruitment to the interviews will be terminated if no new opinions emerge from the data, and this will be defined as no additional material appearing from three consecutive interviews [22]. To achieve the required interview sample it is likely that we will need to invite 30 non-retainers from each trial to allow for an approximate 80% non-response.

#### Data collection

The interview topic guides are informed by the Theoretical Domains Framework (TDF). Whilst the interview guide is informed by the TDF, the interviews will start by exploring personal narratives for trial non-retention and then follow questions based on the TDF. These narratives may identify reasons that do not have a behavioural component and are not be appropriate for intervention, e.g. the direct impact of bereavement. We will develop and refine a topic guide by pilot testing and discussion with the research team (including PPI representatives who will have considerable input into the phrasing of the questions) and trial stakeholders to ensure its comprehensibility, acceptability and theoretical robustness. The number of questions in the interview topic guide will vary for each domain and we will include prompts to address specific constructs within the domains [13]. The interviews will last approximately 20–40 min and will be audio-recorded. A second researcher will listen to the recordings of the first two interviews to check fidelity of the use of the topic guide.

#### Data analysis

Transcribed and anonymised interview transcripts will be imported into NVivo qualitative analysis software version 11 (QSR International, Doncaster, VIC, Australia, 2017) and analysed first through an inductive approach. We will code and compare interview data through a process of constant comparison to provide a summary of the key points that trial participants and trial staff consider important in this context [23]. The themes and the associated data identified from the transcripts will be reviewed by a second member of the project team. We will analyse each group in parallel, but we will also conduct within- and across-group analyses to explore areas of convergence and areas of divergence. A directed content analysis approach will then inform the second deductive stage of analysis to identify behaviours and interactions that are relevant for retention and can be targeted using theoretically informed interventions [24]. Two researchers will code the first three transcripts concurrently to develop a coding strategy, which will be informed by the TDF and the initial inductive analysis. Subsequent transcripts will be coded by one researcher and a random 10% sample will be independently double coded. Inter-rater reliability for coding will be assessed using a Cohen’s kappa calculation with a kappa value of 0.75 or more representing high agreement [25]. Any disagreements in coding will be resolved by a third independent coder, appropriate revisions to the coding strategy implemented, followed by double coding a further 10% until reliability on coding (a kappa of 0.75 or greater) is achieved. We will code the verbatim data to the specific theoretical domains of the TDF using the coding strategy and assessments of their relative importance identified through frequency ranking [26]. Domains will be considered to be ‘relevant’ if frequently mentioned responses determined by scree plot indicate the potential to affect the target behaviour, i.e. trial retention. Also, if domains are low in frequency (< 50%) but codes identify opportunity (variation) to target for change in practice they will also be considered for behaviour change technique (BCT) mapping. We will analyse data on an on-going basis until the target sample size of 45 is achieved or no new responses emerge [22]. The TDF domains identified, and the associated themed transcript data assigned to them, will be reviewed by the second coder to ensure an accurate representation of interview content and to make a judgement about whether the domains meet the criteria for importance (to retention of trial participants) described above.

Initially, we will analyse data from each trial independently so as to allow a nuanced understanding of the specific barriers to retention to be produced. Findings across all trials will then be compared and contrasted to identify areas of both convergence and divergence for trials more generally and also within and across trial participants and trial staff.

### Forming possible solutions (RO3)

Research objectives 1 and 2 will generate the stakeholder data required to inform development of the interventions. Research objective 3 will begin to develop these data into participant-centred, theoretically informed interventions to minimise trial participant drop out.

#### Study design

Identifying intervention components to target barriers and enablers to retention will involve using established methods to map the key theoretical domains (identified in RO2) to behaviour change techniques likely to be effective in addressing those barriers and making use of enablers [12–16]. Further development of the behaviour change techniques into candidate retention interventions will be generated through a co-production workshop, which will be led by both researchers and PPI study members.

#### Mapping of theoretical domains to behaviour change techniques

In brief, we will link the barriers and enablers reported by trial stakeholders to retention-relevant domains of the TDF (RO1 and RO2) which in turn will be used to identify specific behaviour change techniques. This linking of barriers and enablers to domains will be done through the mapping process described by Michie et al. and use a published mapping tool [27]. Three members of the research team will map the relevant TDF domains to the list of 93 BCTs [27]. Coders will independently score whether each BCT could be used to target the TDF domains using a scoring system (see Table 1). As before, inter-rater reliability for coding will be assessed using Cohen’s kappa, with a kappa value of 0.75 or more representing high agreement [25]. BCTs for which there is agreed non-use will be excluded. The remaining BCTs will be ranked by the total count of TDF domains for which there was either ‘agreed use’ and those with the most cross-over will be taken forward for further development.

**Table 1:** Scoring system for choosing potential behaviour change techniques (BCTs) to include in intervention

Decision on inclusion	Scoring for each BCT^a^
Agreed use	Two or more raters scored with a 2 or 3, except if the third rater scored a 0
Agreed non-use	Two or more raters scored with a 0
Disagreement	One rater scored with a 0 and two raters scored with a 2 or 3
Uncertain	All other cells in the matrix

^a^Three raters independently score each BCT as 0–3, where 0 = no, 1 = possibly, 2 = probably and 3 = definitely. Taken from Michie et al. [27]

During the mapping process, the informational content (i.e. ‘what’ will be delivered) and possible modes of delivery (i.e. ‘how’ the behaviour change technique will be delivered) will be considered. Table 2 provides an example of the steps used to choose behaviour change techniques to overcome the barriers and enhance the enablers. The table highlights the focus on ‘technique’, ‘mode’ and ‘content’ as the required elements of intervention development. This process will identify techniques for inclusion in candidate behavioural retention interventions to explore further and prioritise with stakeholders.

**Table 2:** Example of steps used to choose behaviour change techniques for retention interventions

Barriers and enablers that require addressing	Within which theoretical domains do the barriers and enablers operate?	Which intervention components (behaviour change techniques) could overcome the barriers and enhance the enablers?
Low awareness of the trial requirements (i.e. number of follow-up questionnaires) amongst participants.	Knowledge	Technique: Instruction on how to perform a behaviour
		Mode: Patient Information Leaflet, recruitment consultation
		Content: Explicit communication of all follow-up questionnaires (providing personalised timeline of receipt), giving examples of content
Drop out of trial as start to feel well and do not feel the trial ‘monitoring’ (which may have motivated initial participation) is required	Beliefs about consequences	Technique: Salience of consequences
		Mode: Telephone calls by trial team at key time points
		Content: Explaining importance to trial overall of staying in. Explain commitment to trial and why full commitment across the timeline will maximise validity of trial findings, thereby benefiting future patients

#### Co-production workshop to develop candidate retention interventions

##### Participant identification and recruitment

To package the behaviour change techniques as ‘interventions’, we will invite trial non-retainers to participate in a co-production exercise with members of the study team to decide how best to deliver the techniques. By involving trial non-retainers in the co-production of the behavioural interventions we anticipate that the interventions will be more likely to be deemed acceptable and further underpin our participant-centred approach.

We will invite 12 participants (which may include some of those from the interview study in A, and trial non-retainers who were interested to be interviewed but not-required due to sample being full) to participate in the co-production exercise. We will seek expressions of interest in this later stage of the study at the end of the interviews in RO1 or through discussion with those who indicated an interest in interviews but were not included (due to not being required or a suitable time for interview not being identified). We will obtain permission to contact for future linked research during the verbal consent discussion recorded for the interview study. If an adequate number of non-retainers cannot be identified for the co-production activity, this group will be supplemented with trial retainers. We will identify trial retainers through the ongoing trials of the linked clinical trials units (CTUs) whose participants have given previous consent to be contacted about other research or through known contacts of the STEER PPI representatives. We will purposively sample participants so as to maximise diversity but also to include those who will actively and meaningfully engage in the process of co-production. We will send invitation letters to potential participants (again informed by the PPI members) outlining the next stage of the research and asked to contact the STEER research fellow (RN) or either of the PPI members directly if interested in participating in the workshop. Written consent will be sought on the day of the workshop before data collection commences.

##### Data collection

We will design a 1-day workshop with focussed activities, such as demonstrations, role plays and discussions (dependent on the BCTs identified and how they might be incorporated into an intervention), to explore possible options for intervention delivery based on the potential techniques identified from phase B.2. A summary of the findings to date will be generated (by the STEER team) and disseminated to participants 2 weeks before the meeting. Members of the research team (researchers and PPI members) will facilitate the workshop to promote and encourage discussion and observers to document main discussion points. Discussion will be facilitated through a range of prompts and informed by the Affordability; Practicability; Effectiveness and cost-effectiveness; Acceptability; Side-effects and safety; and Equity (APEASE) criteria [28]. The APEASE criteria have been developed for designing and evaluating interventions. The criteria consist of: affordability; practicability; effectiveness and cost-effectiveness; acceptability; side-effects and safety; and equity [28]. All discussions will be audio-recorded where possible. In addition to intervention delivery, if more than one intervention is presented to the co-production team, a prioritisation exercise (e.g. ranking process) will be included. This will be operationalised through the use of an anonymised rating system.

##### Data analysis

We will summarise findings from the workshop and we will agree key points (for each intervention) with any areas of disagreement highlighted. A copy of the findings (structured around the activities conducted on the day of the workshop) will be sent back to workshop participants for agreement or clarification with any additional points being incorporated into the overall findings. These findings will then be fed directly into RO4 of the project, preparing interventions for evaluation.

### Evaluating feasibility and acceptability of the intervention (RO4)

It is likely that many of the trials, for which the developed interventions are applicable, will have outcomes that are collected long after our proposed study ends. Whilst it is unlikely that any of our interventions can be formally evaluated for effectiveness within the timeframe of the current study, an assessment of intervention acceptability and feasibility will be conducted forming an initial evaluation of the developed interventions. In addition, it is critical that the main output from this work is the provision, wide dissemination and initial small-scale implementation of testable intervention packages that can be adopted into ongoing trials, or trials in development. To prepare the ground for rapid future testing of our theory-based retention interventions, we will develop SWAT (Studies Within A Trial) protocols [29]. By developing interventions and presenting them as SWATs, which provide a ready-to-use evaluation framework for others, we will further support collaborative efforts to reduce research waste [30]. Finally, we will develop an explanatory retention framework, informed by the study findings.

#### Study design

##### Intervention acceptability and implementation potential

***Participant identification and recruitment***

We will explore the acceptability of the interventions and their potential for implementation in focus groups with potential trial participants and trial staff. For the purposes of this study, acceptability is defined as ‘*the extent to which people delivering or receiving a retention intervention consider it to be appropriate, based on anticipated or experiential cognitive and emotional responses to the intervention*’ (adapted from Sekhon et al. 2017) [31]. Three focus groups are planned: one with potential trial participants; one with trial staff; and another with Research Ethics Committee (REC) members. We will identify potential trial participants (*n* = 8–10) through the Scottish Health Register for Research (SHARE); we will identify trial staff through the UK Clinical Research Collaboration-registered Clinical Trials Units (CTUs) or UK Trial Managers Network. We will identify REC members through the Health Research Authority distribution lists for REC members. All groups will be invited to participate in a focus group to explore intervention acceptability and potential for implementation. Prospective participants will be sent an invitation letter and study information in the initial email sent from the register or listserv gatekeeper. We will ask interested parties to contact the STEER research fellow (RN) directly (by email) to arrange a convenient time to conduct the focus group. Before the focus group commences the researcher will obtain informed consent, both written and verbal, from participants.

***Data collection***

Discussions will open through presentation of the interventions and will focus discussions on intervention content, mode of delivery and contextual applicability. We will develop the topic guide in collaboration with the STEER PPI members and be informed by the theoretical framework of acceptability (TFA). The TFA is composed of seven constructs: affective attitude, burden, perceived effectiveness, ethicality, intervention coherence, opportunity costs and self-efficacy [31]. Additional questions about potential implementation, barriers and enablers of, will also be included. The STEER research fellow (RN) will facilitate the groups with note-taking and facilitative support from two other members of the research team (KG and a PPI member). The focus groups will be scheduled to last for approximately 1.5 h. Focus groups will be audio-recorded and transcribed. At the end of each focus group we will provide participants with an anonymous questionnaire and ask them to record their extent of agreement with the expressed views of the majority or to state anything that they were not able to voice in the group. This will ensure that those participants who may have disagreed with the majority in the group, but felt unable to voice their disagreement, are given the opportunity to do so (a method used in a previous study conducted by JF).

***Data analysis***

We will code and compare focus group data through a process of constant comparison to provide a summary of the key points about what stakeholders consider important in terms of acceptability and feasibility. We will pay particular attention to any themes arising de novo from the data in addition to a priori-defined component constructs of the TFA and with acknowledgment of the APEASE criteria. We will explore data for confirmatory and dis-confirmatory evidence to support the constructs within the TFA and the APEASE criteria. We will import the transcripts into NVivo and analysed using the Framework approach, which is an established interpretive approach that uses constant comparison techniques [32]. The codes identified, and the associated data assigned to them, from the transcripts will be reviewed by a second member of the project team. Specifically, the analysis will be oriented to address the aim of exploring intervention acceptability with particular attention being paid to implementation of the intervention in practice. We will analyse the data from the questionnaire separately but in a similar method as that described above, paying particular attention to any outliers. Any intervention modifications proposed and agreed during the analysis will be incorporated and included in the SWAT protocols.

##### SWAT protocols

SWAT protocols aim to be brief but specify the requirements for execution of a nested RCT. The SWATs designed in this project will follow existing templates that include details on: background; intervention and comparator; allocation; primary outcome(s); secondary outcome(s); analysis; possible problems; resource requirements and version information [29]. In addition to these pre-specified template headings, we will also incorporate guidance on the more practical aspects of how to embed studies on retention within trials, e.g. providing text to help with ethical approval and considerations for ensuring intervention fidelity. Further to this, the project team will critically reflect on how any change in behaviour (i.e. retention) will be measured and understood, i.e. outcomes and mediators of retention (identified in RO2) and the range of outcomes that may be appropriate for inclusion in the SWAT protocols.

The four trials units represented in this bid alone coordinate approximately 60 trials at any one time, providing a large pool of trials for large-scale testing (outside this proposal) of candidate retention interventions. We will explore implementation of retention SWATs into the procedures of these four trials units during the course of this research – again with learning likely transferable across other units and SWAT design summaries. Each unit will aim to commit to building one of our SWATs into at least one of its trials.

##### Explanatory retention framework

In addition to the SWATs and the exploratory pilot, a retention framework will illustrate potential types of dropout and the contextual factors influencing dropout. This framework will help trialists to identify and think through dropout issues during the design and conduct phases of their trials when they can adjust design, or build in interventions to mitigate potential future problems. This will use the findings from this research project, as well as from recent methodological work on developing SWATs for recruitment strategies and embedding recruitment studies within RCTs [33].

## Discussion

We believe this to be one of the first studies to apply a theoretical lens to the development of participant-centred interventions to improve trial retention. By developing and identifying priority interventions, exploring their acceptability and planning for the evaluation of the interventions in SWATs, this study further supports efforts to reduce research waste [19]. We will track potential effects of trial diversity (e.g. context and trials unit factors) on intervention appropriateness, although we envisage that the lessons from this research will be transferable to other trial contexts.

## Trial status

Trials methodology. All trials that will be involved will be ongoing. The project is registered at www.researchregistry.com (researchregistry2831). A populated SPIRIT Checklist and Figure are available as an additional file (Additional file 1).

## Additional file

Additional file 1: SPIRIT 2013 Checklist completed for STEER Protocol. (DOC 120 kb)

## Abbreviations

APEASE: Affordability; Practicability; Effectiveness and cost-effectiveness; Acceptability; Side-effects and safety; and Equity
BCT: Behaviour change techniques
CTU: Clinical trials unit
MRC: Medical Research Council
PPI: Patient and Public Involvement
RCT: Randomised controlled trial
REC: Research Ethics Committee
RO: Research objective
SHARE: Scottish Health Research Register
SPIRIT: Standard Protocol Items: Recommendations for Interventional Trials
STEER: Systematic Techniques for Enhancing Retention in RCTs
SWAT: Studies Within A Trial
TACTA: Target, Action, Context, Time, Actor
TDF: Theoretical Domains Framework
TFA: Theoretical framework of acceptability
UK: United Kingdom
